# Robotic-Assisted Laparoscopic Management of Vesicoureteral Reflux

**DOI:** 10.1155/2008/732942

**Published:** 2008-08-03

**Authors:** Thomas Lendvay

**Affiliations:** Division of Pediatric Urology, Children's Hospital and Regional Medical Center, University of Washington, 4800 Sand Point Way, NE, Seattle, WA 98102, USA

## Abstract

Robotic-assisted laparoscopy (RAL) has become a promising means for performing correction of vesicoureteral reflux disease in children through both intravesical and extravesical techniques. We describe the importance of patient selection, intraoperative patient positioning, employing certain helpful techniques for exposure, and recognizing the limitations and potential complications of robotic reimplant surgery. As more clinicians embrace robotic surgery and more urology residents are trained in robotics, we anticipate an expansion of the applications of robotics in children. We believe that it is necessary to develop robotic surgery curricula for novice roboticists and residents so that patients may experience improved surgical outcomes.

## 1. BACKGROUND

The minimally invasive surgical (MIS) approach to vesicoureteral reflux disease was first described by Atala et al. in minipigs in 1993 and then first described in humans by Ehrlich et al. in 1994 [1, 2]. Since then, few pediatric centers have embraced either the laparoscopic extravesical or vesicoscopic cross-trigonal approaches owing to the technical challenges of fine suturing in the small spaces. Success rates have been comparable to open surgical techniques and in 2004; Peters described his experience using the surgical robot as an adjunct to both transvesical and extravesical repairs [3]. Since then, urologists have watched robotic surgery becoming the standard of care in some adult urologic procedures such as radical prostatectomy, but application in pediatrics has been limited to a few centers where the robot has been accessible to pediatric urologists.

The surgical robot allows clinicians improved dexterity, three-dimensional visualization, and motion scaling, which helps dampen physiologic tremor.** Due to these benefits, the reconstructive techniques required for ureteral reimplantation are well suited for robotic surgery. In addition, due to the enhanced learning curve with robotic surgery over pure laparoscopy, surgeons are able to utilize the same techniques and suture size as would be used in open surgery. Major advantages over pure laparoscopic and open techniques are 10X visual magnification and three-dimensional visualization, and the ergonomic considerations of the robot console where the surgeon sits during the procedure. The limitations of robotic surgery are the added cost to the host institution, the increased operative times required, and the support required from the ancillary operative staff. Interestingly, these limitations are the same experienced by the initial laparoscopists of the 90s.

The key aspects of successful robotic ureteral reimplantation surgery include appropriate patient selection, proper patient positioning, an armamentarium of helpful techniques to facilitate exposure, and an understanding of the limitations of the robot and the complications potentially encountered.

## 2. PATIENT SELECTION

In counseling our patients for the options of surgical correction of vesicoureteral reflux, we rely heavily on the individual patient's clinical picture.** All patients are offered both endoscopic and formal surgical repairs, whether by minimally invasive or open techniques. We detail peer-reviewed cited and personal success experience for our patients and inform them of the variations in success that can be expected in the face of higher grades of reflux and voiding dysfunction. It is difficult to generalize or standardize patients, but typically, formal surgical repairs are reserved for patients with higher grades of reflux, severe voiding dysfunction, or in those with duplex systems. Patients with lower grades of reflux may be more appropriate for intramural ureteral bulking agent implantation. When discussing robotic/laparoscopic techniques versus open surgical techniques, we highlight the fact that open surgery is the “gold-standard,” and MIS repairs appear to have similar success rates as open surgery. Since we do not discern pure laparoscopy from robotic-assisted laparoscopy because we believe that the robot is merely another adjunct or tool to laparoscopy, we only describe that we use the robot to assist with reconstructive surgeries.

Patient comorbidities have not played a major role in the decision for robotic repairs, however, patients with severe pulmonary reserve deficits need to be carefully evaluated preoperatively by anesthesia to determine if abdominal insufflation may impair ventilation. In addition, children with prior abdominal surgery may require additional dissection in the abdomen to lyse any adhesions that may obscure the line of sight to the pelvis.

In our experience, patient's size has not limited our decision for robotic surgery in part because it is unusual to operate on children less than 6 months of age for vesicoureteral reflux and because we have not found that the intuitive working port-to-camera port distance recommendations of 8–10 cm to be applicable in small children. We have successfully used interport distances of 5 cm without any arm collisions. We believe that this is due to the small operative field and few large arm movements required once the robot is appropriately set up and docked.

## 3. PATIENT POSITIONING

As with all robotic surgeries in children, appropriate patient positioning is critical to the efficient progression and success of the case. Since it is our practice to perform cystourethroscopy prior to ureteral detrusorraphy surgery, we place the patient in a low lithotomy position and prep the patient for both cystoscopy and laparoscopic access at the same time. We angle the patient in 10 degree Trendelenberg to encourage the bowel to fall out of the pelvis. For bilateral repairs, we choose to place indwelling stents if the child has a history of a trabeculated thickened bladder due to voiding dysfunction as we have observed postoperative edema at the neotransmural tunnel causing transient obstruction. For the majority of cases, we typically will place external ureteral catheters attached to a urethral catheter to help guide ureteral dissection during the procedure. These are removed at the end of the surgery.

**Figure 1:**
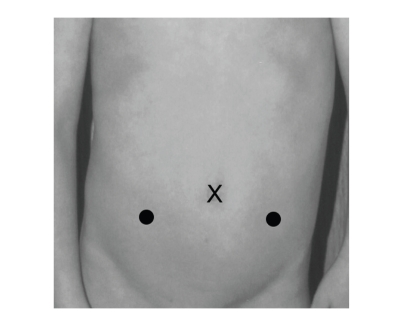
Port placement, bilateral reimplants. X = camera port, black dots = working ports.

**Figure 2:**
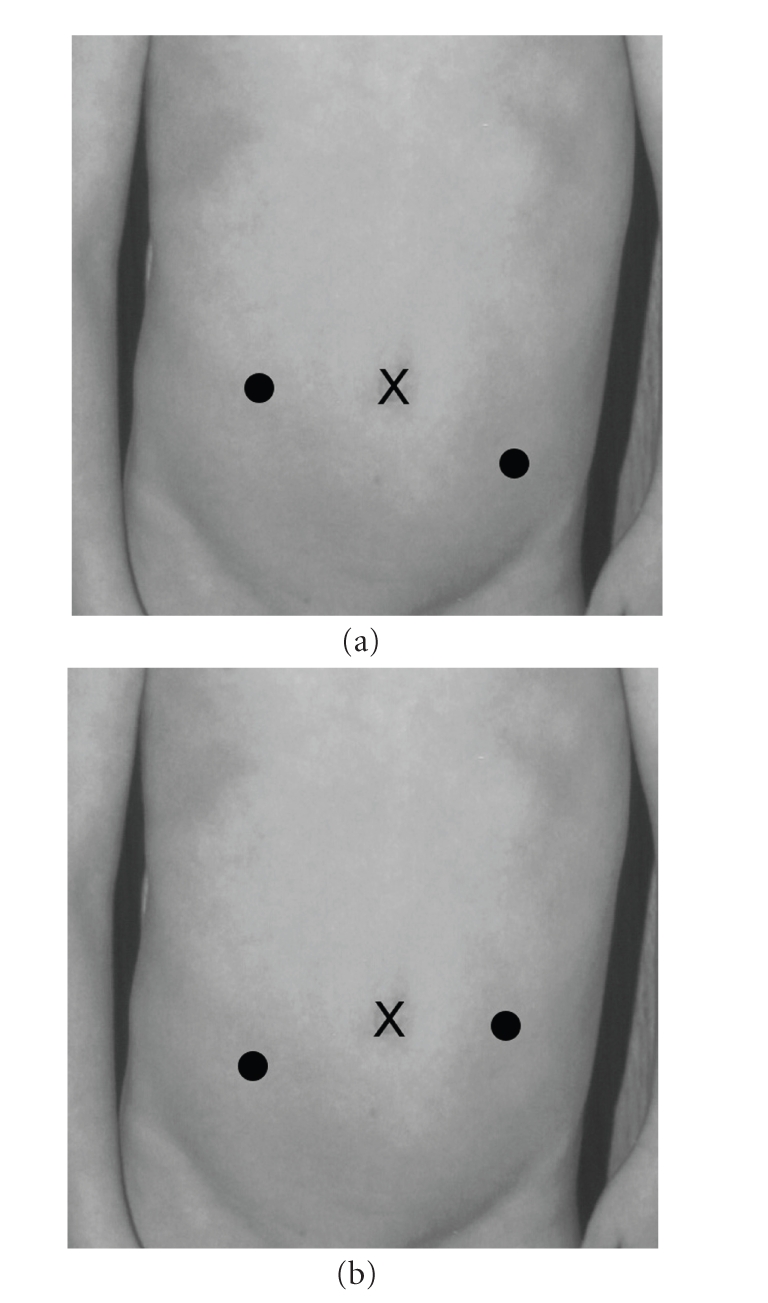
(a) Port placement, right reimplant; (b) port placement, left reimplant.

**Figure 3:**
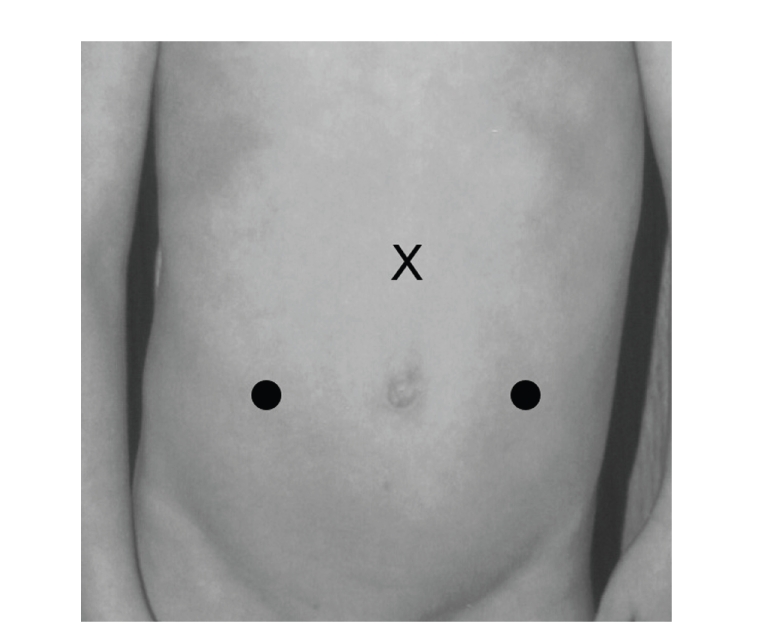
Port placement, small children or short-waisted children, bilateral reimplants.

Although some institutions have used the vesicoscopic approach for ureteral reimplant surgery [4, 5], we use a transperitoneal approach because we find that working spaces are not limiting and we are more comfortable with this approach. We use a two-armed robot and place the camera port through the umbilicus. The two working ports are placed at the paramedian lines slightly below and on either side of the umbilicus to avoid the inferior epigastric vessels (Figure 1). In children less than 15 kg, we have tended to place the working ports at the level of the umbilicus to ensure a good distance to the target site. For bilateral cases, the robot is situated at the patient's feet in the midline; however, for unilateral repairs we position the robot at the ipsilateral foot. In addition, the ipsilateral working port is placed slightly higher than the contralateral working port (see Figures 2(a), 2(b)). In small infants, we place the camera port subxyphoid, to ensure a good working distance of the camera to the target site (Figure 3).

At our institution, we have found that the most efficient way to set up our robotics room is with a fixed location for the console and a relatively fixed location for the robot. We move the patient bed, the video cart, and the instrument table depending on the access we desire. For reflux surgery, we position the tower on either side for bilateral repairs or on the ipsilateral side for unilateral repairs. This allows for easy access for the bedside assist and scrub tech to be on the contralateral side with the instruments. In the event that pure laparoscopy maneuvers are necessary, there is ample room.

## 4. INTRAOPERATIVE TRICKS

**Figure 4:**
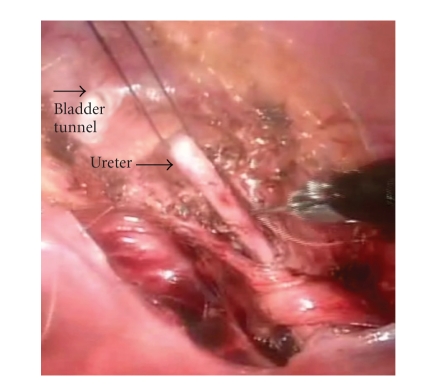
Demonstration of *hitch stitch* around right ureter for retraction (2-0 monofilament).

There are certain maneuvers which are unique to laparoscopic/RAL surgery which assist in expediting the surgeries and allow for the minimum number of ports to be placed. A sharp criticism of minimally invasive surgery in children, especially small children, has been that open surgery incisions are not as morbid as in adults and that the additive incisional length of minimally invasive surgeries may equal and sometimes exceed the total length of a single open surgical incision thereby theoretically causing more postoperative pain. This argument is flawed because Blinman has demonstrated that the sum tensions of port incisions do not equal the whole incisional tensile burden as conjectured by some open surgeons [6]. We believe that the smallest and fewest possible ports should be used to safely and effectively perform MIS surgery, therefore, we employ the use of hitch-stitches to assist in organ retraction throughout our cases [3]. During an extravesical ureteral reimplant, we routinely use monofilament suture placed through the lower abdominal wall to aid in retraction of ureters and the bladder (Figure 4). During creation and closure of the detrusor bladder flaps, we find that a hitch stitch to help elongate the bladder anteriorly ensures proper length and straightening of the tunnel. In addition, we use anteriorly retracted stitches around the ureters to assist in laying the ureters down in the detrusor tunnels. To lessen constant tension on the ureter with this stitch, we routinely release the tension from outside of the abdomen when retraction is not needed. When no longer needed, these sutures are removed leaving only behind small needle puncture marks on the suprapubic skin. For children with more subcutaneous fat, we lengthen the hitch stitch needles by partially flattening them (skiing).

Throughout the creation of the detrusor tunnel and the detrusorotomy, we intermittently insufflate the bladder through the indwelling urethral catheter with a second insufflation unit to ensure appropriate position of the ureter as described by Yeung et al. [7]. We have used both manual fluid bladder instillation for distention and gas insufflation and have found the gas to be more rapid in raising and dropping the bladder and in the event of a small mucosotomy which would require oversewing, the liquid distention tends to make for a more tedious closure.

## 5. COMPLICATIONS

With the adoption of new techniques, we have experienced some complications which can be attributed to developing familiarity with minimally invasive reimplant surgery. When counseling families about the adverse outcomes of ureteral reimplant surgery, we discuss urine leak, urinary obstruction, and urinary retention. Casale et al. have published their series of 41 bilateral extravesical RAL reimplants without any post-op urinary retention. **They attribute the absence of retention, despite open surgical extravesical repair literature citing up to 10% postoperative retention, due to the improved visualization of the neurovascular bundle that is situated just lateral to the ureteral hiatus [8, 9]. On the other hand, Peters encountered postoperative voiding dysfunction in his experience of extravesical robotic reimplants [3]. We have had only one patient who had mild retention post-op and we anticipated this because of his preoperative urinary retention history so we placed a percutaneous suprapubic tube at the time of his reimplant surgery for postvoid drainage.** His tube was removed 2 weeks later after his retention improved to less than 10% of his functional capacity.

Early in our experience, we had an adolescent female present one week postoperatively with unilateral labial swelling and abdominal pain. She was found to have a unilateral ureteral leak just outside of the neohiatus and required temporary stenting. The leak sealed and her reflux was successfully treated confirmed by VCUG. It is possible that electrothermy dissection near the ureteral insertion to the bladder may have caused this leak and since then, only nonenergy dissection is used to raise detrusor flaps near the ureteral hiatus. Another child with severe elimination syndrome and a thickened bladder wall who underwent bilateral ureteral reimplants developed transient postoperative ureteral edema leading to azotemia. She required temporary ureteral stenting after which her azotemia resolved. VCUG and US after stent removal confirmed successful reflux resolution and no ureteral obstruction. In lieu of this outcome, we also advocate stenting of children with solitary kidneys to avoid the possibility of postoperative transient acute renal failure as recommended by Peters [3].

As described by Casale et al., we have encountered the uterine artery in our female patients [9]. During open extravesical reimplants, the uterine artery is rarely identified, but with abdominal insufflation, the bladder is situated anteriorly in the operative field thereby giving the appearance that the ureter must be stretched to lay down in the detrusor trough. The uterine artery will appear to kink the ureter or press it posteriorly as it is laid down in its detrusor trough. During abdominal desufflation, however, one will see that the kinking is merely an artifact of the distention.

Beyond these early complications, we have not witnessed subsequent urinary retention, urine leak, or ureteral stenosis as identified by any *de novo* hydronephrosis. In addition, we have had three VCUG-documented reflux management failures or reflux down grades and one case of *de novo* contralateral reflux out of 16 patients. All failures have been in children with varying reflux grades and the only common element of these children has been a history of pre-existing elimination syndrome, a factor well known to reduce antireflux surgery success.

## 6. FUTURE PERSPECTIVES

Initial reports of the success of RAL reimplant surgery seem to rival open surgical repairs. To date, MIS surgery in children has demonstrated equivalence to open surgery with additional cost. The advantages of robotic surgery in children are harder to demonstrate than in adults since metrics used in the adult literature to show advantages do not always apply to children. The financial cost from loss of work productivity is more measurable than the impact of missed days of school. In addition, few have looked at the financial impact of a working parent having to stay at home to care for a postoperative child. Pain score assessments between open and MIS surgeries have not been rigorously tested as randomized trials looking at open versus robotic urologic surgeries in children are nonexistent. Formal multi-institutional prospective studies looking at matched open and RAL VUR patients are required.

**Figure 5:**
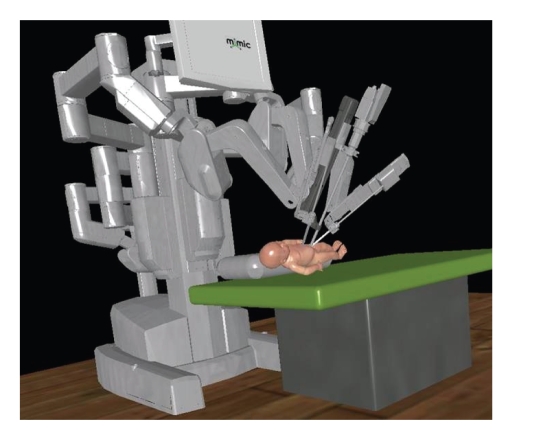
Patient-specific virtual reality robot docking simulation (Courtesy of MIMIC Technologies, Inc., Seattle, WA, USA).

The advantages for advancing robotics in children will be the greatest in residency education and patient-specific surgical simulation (Figure 5). With the aid of preoperative imaging, a surgeon or resident will be able to perform the surgery in a virtual reality arena prior to performing the surgery on the actual child. MIS surgery lends itself to task deconstruction better than open surgical procedures and we believe that in the era of surgical simulation training, robotic surgery will allow residents and novice roboticists to acquire technical competence in procedure performance more rapidly than open surgical procedures. The development of robotic surgery curricula will be necessary to achieve the highest level of patient outcomes.
